# Complex intestinal fistula treatment and care: A case report and literature review

**DOI:** 10.1097/MD.0000000000040511

**Published:** 2024-11-15

**Authors:** Yuee Hu, Yanyan Qin, Wei Dong, Yuxu Zhong, Haibo Chu

**Affiliations:** a Department of General Surgery, Jiaozhou Branch of Shanghai East Hospital, Tongji University, Qingdao, China; b State Key Laboratory of Toxicology and Medical Countermeasures, Institute of Toxicology and Pharmacology, Beijing, China.

**Keywords:** abdominal cocoon, intestinal fistula, nursing, perioperative period, traumatic intestinal rupture

## Abstract

**Rationale:**

Abdominal cocoon is an uncommon abdominal disease. Intestinal rupture complicated with intestinal fistula rarely occurs in patients with abdominal cocoon.

**Patient concerns:**

A 51-year-old man was referred to hospital, with a 4-hour history of abdominal injuries caused by traffic accident. Intraoperatively, the small intestine in the abdominal cavity was surrounded by dense, tough, grayish-white fibrous tissue. There were the rupture of 2 sites in the ileum. The ileum was anastomosed side-to-side using a cutting and closing device. The patient was postoperatively transferred to the intensive care unit and received ventilator-assisted breathing, along with anti-infection and supportive treatments. On the 10th day after surgery, grass green turbid fluid of approximately 150 mL was extracted from the abdominal drainage tube. The secondary laparotomy was performed on the 12th day post-surgery, revealing a 1.5 cm diameter fistula at the end of the ileum.

**Intervention and outcomes:**

Nursing strategies included ensuring optimal mechanical ventilation for oxygenation, utilizing Li’s double cannula for continuous abdominal irrigation and negative pressure drainage to prevent abdominal abscess formation, emphasizing the importance of enteral nutrition, implementing direct suture treatment to manage retrograde infection and expedite stoma healing, and employing Li’s double cannula and vacuum-assisted closure technique to promote incision healing. After 48 days secondary post-surgery, the incision was fully healed, and the patient was discharged home with the stoma bag. Five months later, he was readmitted to the hospital, and the stoma was reversed.

**Lessons:**

Intestinal fistula poses a remarkable challenge after abdominal cocoon surgery, typically manifesting 4 to 5 days postoperatively. However, in this case, it occurred on the 10th day, highlighting the critical role of vigilant monitoring of drainage fluid color and volume in postoperative care. Navigating the complex management of intestinal rupture in abdominal cocoon necessitates a more efficacious approach, highlighting the importance of accumulating comprehensive nursing expertise through such cases.

## 1. Introduction

Abdominal cocoon, also known as idiopathic sclerosing peritonitis, is a rare abdominal disease. It is characterized by dense fibrous membranes covering partly or entirely the small intestine and abdominal organs.^[1]^ The etiology of abdominal cocoon remains elusive, its preoperative diagnosis is difficult, and its misdiagnosis rate was as high as 95.4%.^[2]^ The average mortality rate for abdominal cocoon disease was reported to be 35%, and more than 60% of patients were identified with severe disease.^[3]^ In 1978, Foo initially defined the condition as an abdominal cocoon, categorizing it into primary and secondary types. It is more frequently found in tropical and subtropical countries.^[4]^ It is more frequently identified in men, and the male-to-female ratio for this disease was approximately 1.2 to 2:1.^[5,6]^ Primary abdominal cocoon is caused by curled embryo, abnormal differentiation of mesoderm, and abnormal development of dorsal mesentery during the embryonic period of intestine. It is mainly accompanied by the absence of omentum, the absence of gastrocolic ligaments, malrotation of bowel or colon, visceral transposition, cryptorchidism, hernia, and other diseases.^[7]^ Secondary abdominal cocoon includes pelvic infections, a history of abdominal surgery, long-term peritoneal dialysis, malignant tumors, abdominal tuberculosis, autoimmune diseases, liver transplantation, abdominal trauma, hepatitis C, chemotherapy drugs, β-blockers, mercury, etc.^[8,9]^ Abdominal cocoon is divided into 3 clinical types: type I, partly involving the small intestine; type II, entirely involving the small intestine; and type III, entirely involving the small intestine and extending to other organs.^[10]^ Clinically, incomplete or complete intestinal obstruction and abdominal mass are more common manifestations,^[11,12]^ while intestinal perforation and fistula rarely occur.^[13,14]^ It has been frequently demonstrated that abdominal cocoon could result in hollow organ rupture due to car accidents.^[15–17]^ However, cases of abdominal cocoon resulting from small intestine rupture due to car accidents have been rarely reported. This study aimed to report the nursing experience of a case involving an abdominal cocoon with an intestinal fistula following ileal rupture caused by a car accident.

## 2. Case presentation

### 2.1. General information

**Figure 1.**
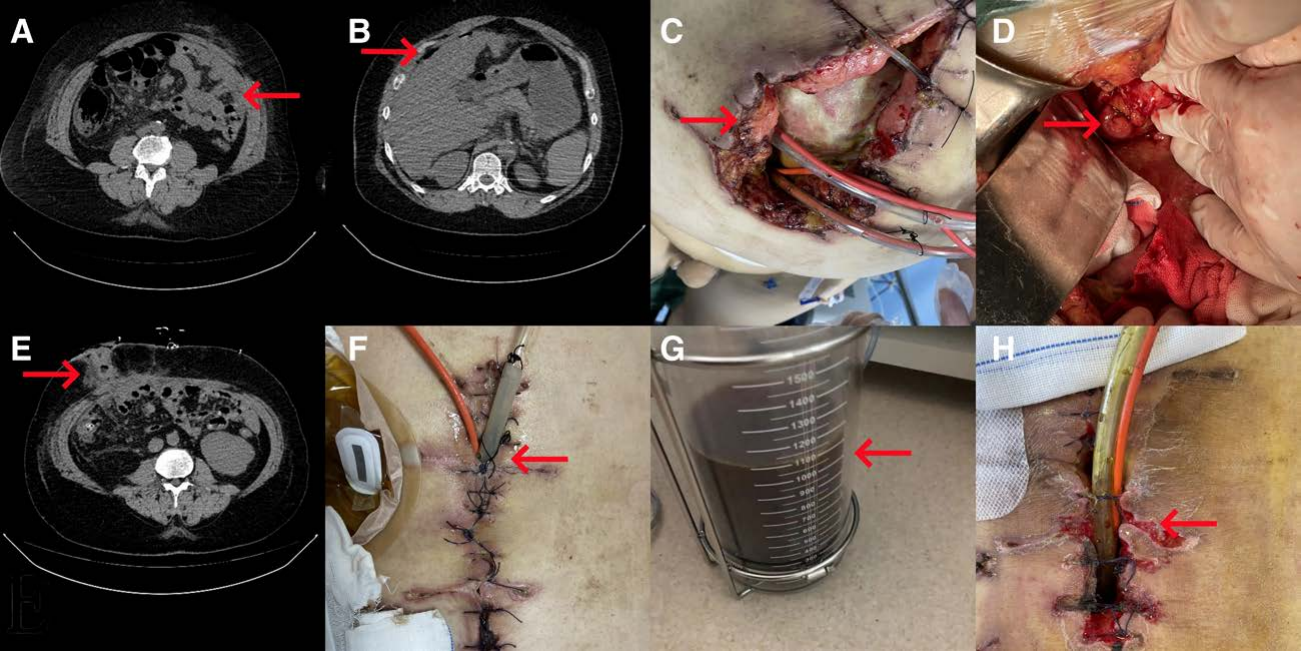
Computed tomography (CT) images and incision changes. (A) Abdominal CT revealed dense, clumped images in the abdominal wall, with some small intestinal loops gathered, indicative of the cauliflower sign. (B) Subphrenic free air was observed. (C) Postoperative abdominal infection and incisional tearing were evident. (D) Intraoperative ileal anastomotic fistula was identified. (E) Abdominal CT displayed a single ileal stoma. (F) The incision was sutured, and Li’s double cannula was placed subcutaneously. (G) Drainage from the incision exhibited turbid bloody fluid. (H) Silk cutting and necrosis of the skin margin in the incision were evident (marked by the red arrow).

A 51-year-old man was referred to Jiaozhou Branch of Shanghai East Hospital, Tongji University (Qingdao, China) on June 27, 2022, with a 4-hour history of abdominal injuries caused by traffic accident. The patient had no prior history of abdominal pain. Physical examination revealed the following outcomes: body temperature of 36.8 °C, heart rate of 129 beats/min, respiratory rate of 23 breaths/min, and blood pressure of 90/60 mm Hg. The patient was conscious, and had a fat body type, weighing 124 kg, and was in a passive position. He presented with a soft tissue avulsion of the left nasal wing, tenderness, and tenderness in the right chest wall. The patient also exhibited abdominal distension, abdominal tenderness, rebound pain, muscle tension, and absence of bowel sounds. A soft tissue avulsion of about 15 × 8 cm^2^ was found in the anterior tibial area of the right calf, which was deepened into the muscle layer. Laboratory tests showed the following outcomes: leukocyte count, 8.07 × 10^9^/L; neutrophil count, 88.7%; hemoglobin (Hb) level, 99 g/L; C-reactive protein level, 5.78 mg/L; albumin (ALB) level, 32 g/L; alanine transaminase level, 36.7 U/L; cholinesterase level, 3525 U/L; creatinine level, 93 µmol/L; and urea nitrogen level, 5.20 mmol/L. The patient’s electrocardiogram revealed sinus tachycardia. Chest computed tomography (CT) scan showed fractures of the right 6th, 7th, and 8th ribs, many flaky shadows of the right lung, and gas–liquid level of the right thoracic cavity. Abdominal CT scan revealed a dense mass in the abdominal wall and several loops of the small intestine clustered together, presenting a cauliflower sign. Additionally, there was free gas in the abdominal cavity and a small amount of perihepatic fluid (Fig. 1A and B). Pelvic CT scan showed multiple fractures of the pelvis.

### 2.2. Final diagnosis

Upon admission, the diagnosis included severe multiple injuries and traumatic shock: intestinal rupture and acute peritonitis, abdominal wall grinding contusion and subcutaneous hematoma, fractures of the right 6th, 7th, and 8th ribs, hemopneumothorax, and traumatic wet lung, multiple fractures of the pelvis, facial soft tissue avulsion injury, and soft tissue avulsion injury of the right calf.

### 2.3. Treatment

The 1st stage of treatment involved emergency surgery and partial ilectomy (1 day after admission): an emergency laparotomy was performed. Intraoperatively, a large separation was found between the fascia of the anterior abdominal wall, with significant accumulation of blood clots. The small intestine in the abdominal cavity was surrounded by dense, tough, grayish-white fibrous tissue. Upon incision, approximately 1000 mL of yellow-green cloudy liquid and food residue were found. There was a 3 × 2 cm^2^ rupture in the ileum, 30 cm from the ileocecal part, with additional ruptures measuring 2 × 1 cm^2^ at 35 and 45 cm away. The peritoneal fluid was absorbed, the adhesive intestinal loops were separated, and segments of the ileum measuring 14 and 4 cm were excised. The ileum was anastomosed side-to-side using a cutting and closing device. The abdominal cavity was repeatedly washed with a large amount of warm normal saline, followed by hydrogen peroxide and dilute iodophor saline. Three drainage tubes were placed, and the abdomen was closed. Debridement and suturing of the nasal alar and right calf were carried out by the otolaryngology and orthopedics departments, respectively.

The 2nd stage of treatment involved life-supporting and making an intestinal fistula diagnosis (10 days after admission): the patient was postoperatively transferred to the intensive care unit (ICU) and received ventilator-assisted breathing, along with anti-infection and supportive treatments. On the 9th day after surgery, the ventilator was removed and the tracheal tube was pulled out. On the 10th day after surgery, the body temperature remained as high as 39 to 40 °C, and grass green turbid fluid of approximately 150 mL was extracted from the abdominal drainage tube. Laboratory tests indicated the following results: leukocyte count, 16.4 × 10^9^/L; neutrophil count, 90.4%; Hb level, 83 g/L; alanine transaminase level, 67.8 U/L; cholinesterase level, 2756 U/L; ALB level, 28 g/L; pre-ALB level, 0.1 g/L; creatinine level, 125 µmol/L; urea nitrogen level, 7.63 mmol/L; interleukin-6 level, > 5000 pg/mL; and procalcitonin level, 42.27 ng/mL. Bedside ultrasound revealed bilateral pleural effusion, with a perihepatic effusion thickness of 1.5 cm and a right lower abdominal small intestine effusion thickness of 2.2 cm. Retrograde angiography through the drainage tube indicated the entry of contrast agent into the ileum, suggesting an intestinal fistula. Consequently, a Lee’s double cannula was inserted into the abdominal cavity for continuous irrigation and negative pressure drainage.

**Figure 2.**
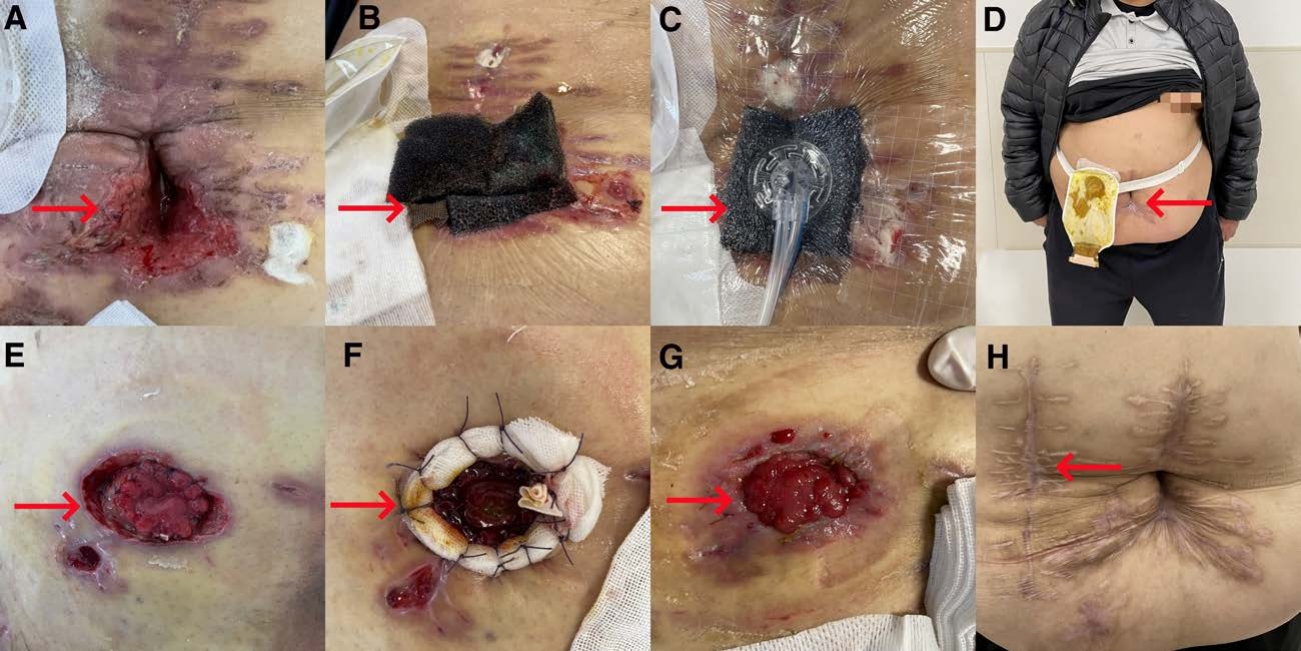
Incision changing and stoma separating. (A) Removing sutures and opening incisions. (B) Incisions were filled with sterile medical sponges. (C) The vacuum-assisted closure technique was used to promote granulation tissue growth. (D) Incision healing. (E) The stoma was separated and intestinal fluid spilled into the subcutaneous. (F) The separated stoma was sewn with 4-0 absorbable thread, and then covered with a rubber sheet and Vaseline gauze. (G) After 14 days, sutures around the stoma were removed, and healing occurred between the intestinal wall and the abdominal wall. (H) The stoma was reconnected 5 months postoperatively (marked by the red arrow).

The 3rd stage of treatment involved the secondary laparotomy and Li’s double cannula draining (11–23 days after admission): On the 11th day post-surgery, the abdominal incision split was opened, revealing a significant amount of purulent secretion in the abdominal cavity. Two Li’s double cannulas were then placed for continuous irrigation and negative pressure drainage. The drainage fluid reached 600 mL/24 hours, appearing as grass green turbid fluid (Fig. 1C). Peritoneal drainage fluid and blood culture indicated the growth of *Escherichia coli*, prompting a change in antibiotics for sensitization, along with reinforced anti-infection and supportive therapy. The secondary laparotomy was performed on the 12th day post-surgery, revealing a 1.5 cm diameter fistula at the end of the ileum, 15 cm from its terminus (Fig. 1D). Inflammatory “frozen” adhesions were observed between the intestinal tubes in the abdominal cavity, and the fascia space of the abdominal wall was separated by stealth. After draining the abdominal fluid, the abdominal cavity was repeatedly irrigated with 6000 mL of warm saline. The intestinal tubes were dissociated using ultrasonic and electric knives, and a segment of the ileum measuring 5 cm was excised using a cutting and closing device. The distal end was then closed (Fig. 1E). The subcutaneous tissue of the incision was dissected, and the peritoneum and muscle layer were discontinuously sutured using 3-0 absorbable sutures. The subcutaneous tissue was irrigated with normal saline and dilute iodophor saline, and a Li’s double cannula was inserted before discontinuous skin suturing (Fig. 1F). On the 1st day after the secondary surgery, the patient’s vital signs were within normal limits, with a body temperature of normal, pulse rate of 91 beats/min, respiratory rate of 18 breaths/min, and blood pressure of 105/54 mm Hg. The patient was conscious, extubated, and the tracheal cannula was removed. By the 3rd day after surgery, the patient’s urine output was 2500 mL/day, pulse rate decreased to 62 beats/min, respiratory rate was 12 breaths/min, and blood pressure was 152/92 mm Hg. When the patient’s condition was relatively stable, it was attempted to transfer him to the gastrointestinal surgery department. Treatment strategies included maintaining systolic blood pressure below 120 mm Hg to reduce bleeding in the abdominal cavity and incision, continuous gastrointestinal decompression, administration of somatostatin to relieve abdominal pressure, and anti-infective therapy. A Li’s double cannula was utilized for discontinuous irrigation and negative pressure drainage, yielding cloudy bloody fluid from the abdominal incision (Fig. 1G). By the 5th postoperative day, the incision appeared dry and clean without any secretions, prompting removal of the Li’s double cannula and initiation of enteral nutrition. However, on the 8th day after surgery, increased exudation and secretions were found, requiring repositioning of the Li’s double cannula for intermittent irrigation and continuous negative pressure drainage. By the 12th day post-surgery, silk cutting and necrosis of the incision’s skin margin were evident (Fig. 1H). Sutures were removed, and the vacuum-assisted closure technique was applied to promote granulation tissue growth (Fig. 2A–C).

**Figure 3.**
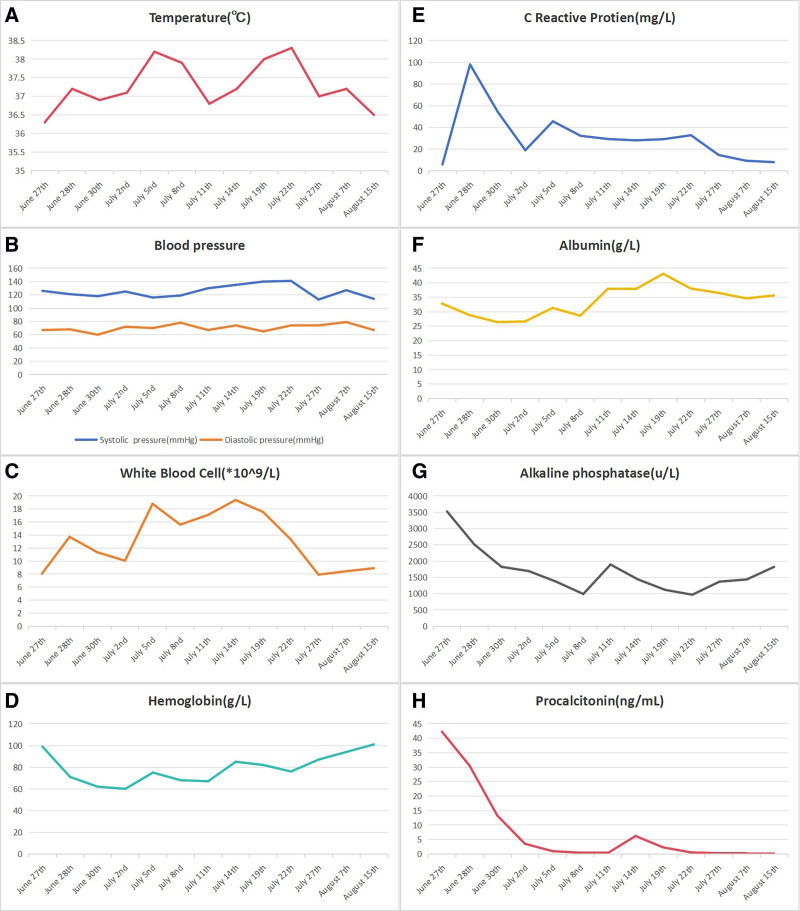
Trend charts of the patient’s vital signs and hematological parameters. (A) Dynamic changes in body temperature. (B) Dynamic changes in blood pressure. (C) Dynamic changes in white blood cells. (D) Dynamic changes in C-reactive protein. (E) Dynamic changes in hemoglobin. (F) Dynamic changes in cholinesterase. (G) Dynamic changes in cholinesterase. (H) Dynamic changes in procalcitonin.

The 4th stage of treatment involved treating separation of the stoma and enteral nutrition (24–48 days after admission): On the 13th day after surgery, the patient developed fever, and separation of the stoma (3/5 circumference) was noted, allowing intestinal fluid to flow back into the subcutaneous space (Fig. 2E). The stoma was re-sutured with interrupted 4-0 absorbable sutures and surrounded by Vaseline gauze strips (Fig. 2F). Enteral nutrition was suspended, somatostatin was administered, and an abdominal compression bandage was applied to prevent incisional dehiscence. After fasting for 5 days, enteral nutrition was resumed, and somatostatin was discontinued. The wound on the right calf was managed using the vacuum-assisted closure technique with continuous irrigation and negative pressure drainage. On the 17th day after surgery, body temperature was normal for 2 days, white blood cell count and neutrophil count were both normal, and antibiotics were discontinued. On the 25th day after surgery, enteral nutrition was stopped and change to liquid diet. The patient’s vital signs and hematological parameters were gradually stabilized (Fig. 3A–H).

Finally, the 5th stage of treatment involved small intestinal fistula recovery: On the 27th day after surgery, the oil gauze around the stoma was removed, and the intestinal wall and abdominal wall were healed (Fig. 2G). After 48 days post-surgery, the incision was fully healed, and the patient was discharged home with the stoma bag (Fig. 2D). Five months later, he was readmitted to the hospital, and the stoma was reversed (Fig. 2H). Informed written consent was obtained from the patient’s wife for publication of this case report and the accompanying images. The reporting of this study conforms to the CARE guidelines. The study was approved by the Jiaozhou Branch of Shanghai East Hospital, Tongji University Ethics Committee (approval number: JZBSEHTJ-2024-012; approval date: March 6, 2024).

## 3. Discussion

### 3.1. Mechanical ventilation management

In ICU treatment, precise mechanical ventilation management can effectively stabilize patients’ vital signs. Nursing interventions should be targeted to reduce the incidence of ventilator-associated pneumonia in ICU patients. Nursing interventions should be precisely adjusted based on variations in ventilator modes and parameters, as well as the patient’s specific clinical status. Emphasis should be placed on optimizing oxygen delivery and minimizing oxygen consumption to effectively restore the patient’s respiratory function.^[18,19]^ The nurse should adhere strictly to the scheduled sputum aspiration procedure and ensure that the suction catheter extends beyond the inner opening of the tracheal intubation tube. This precaution is essential to prevent sputum accumulation at the inner opening of the tube, which can compromise mechanical ventilation efficacy and increase the likelihood of lung infections. For patients with thick sputum, continuous airway humidification should be given to dilute sputum aspiration. Patients with anxiety and asthma should collaborate closely with physicians for appropriate management. Given the presence of multiple postoperative tubes in the present case, nurses must document the nature and quantity of gastric fluid, drainage, and urine over a 24-hour period. Precise control of fluid intake is essential to prevent excess, thereby reducing the risk of pulmonary edema and right heart failure. Furthermore, patients in this stage experience heightened catabolism due to trauma and infection, compounded by mechanical ventilation, resulting in substantial energy depletion. Therefore, timely administration of heat and albumin supplements is critical to stabilize the patient’s condition and address hypoproteinemia effectively.

### 3.2. Nursing of intestinal fistula

It has been reported that intestinal fistula in patients with abdominal cocoon frequently occurs 4 to 5 days after surgery.^[4]^ In this case, it was found that the drainage fluid was grass green at 10 days after surgery, and its amount gradually increased. The retrograde angiography of the drainage tube revealed that the contrast agent had entered the intestinal cavity. In the event of an intestinal fistula, a significant volume of intestinal fluid may overflow into the abdominal cavity, promoting rapid bacterial proliferation and increasing the risk of abdominal infection.^[20]^ In this case, the patient had intestinal fistula, peritoneal *E coli* infection, sepsis, and hypoproteinemia. The ordinary drainage tube was replaced with Li’s double cannula for continuous irrigation and negative pressure drainage in time. The total volume of irrigation fluid reached 6000 mL per day, with a maintained irrigation rate of 60 to 70 drops per minute and a negative pressure of 15 kPa. The rate of irrigation fluid was adjusted based on the color and characteristics of the drainage fluid. Literature reports caution against excessive irrigation speed, which could impede suction and lead to fluid accumulation, increasing the risk of infection. Conversely, slow irrigation may result in dry suction, causing bleeding and inadequate drainage.^[21]^ The incision was breached on the following day, prompting the placement of 2 Li’s double cannulas for continuous irrigation and negative pressure drainage, minimizing the need for frequent dressing changes. Full abdominal cavity drainage reduces toxic blood absorption, alleviating the patient’s clinical symptoms (chills, high fever, lethargy). The cross-helix method secured Li’s double cannulas to the abdominal wall, limiting tube length to prevent entanglement and ensuring proper positioning to prevent dislodgment. Adjustment of drip pipe and double cannula positions during position changes maintains continuous and effective suction. Daily monitoring and recording of drainage fluid volume and characteristics maintain fluid balance. The assigned nurse should check the suction sound of the abdominal drainage tube every 2 hours, simultaneously compressing the tube to prevent ineffective suction and drainage.

### 3.3. Nutritional support nursing

#### 3.3.1. Nutritional assessment

The patient’s height was 166 cm, weighed 124 kg, and his body mass index was 45 kg/m^2^. Postoperatively, the patient’s ALB level was 28 g/L, pre-ALB level was 0.1/L, and Hb level was 83 g/L. According to the Nutrition Risk Screening 2002, with a total score of ≥ 3 points, the patient was categorized as high nutritional risk and required early nutritional support as follows: ① severe multiple injuries and traumatic shock (intestinal rupture, right hemopneumothorax and traumatic wet lung, and pelvic fracture) 1 score; ② major abdominal surgery 2 scores; ③ intestinal fistula, sepsis, intensive care patients (APACHE > 10 scores) 3 scores; ④ within 1 month (weight loss > 5%, serum albumin < 35 g/L) 3 scores; total score was 9 scores.

#### 3.3.2. Nutritional pathway selection

On the basis of abdominal cocoon, the patient suffered from bruising in abdominal wall and crushing injury in abdominal cavity caused by intestinal rupture due to car accident. It was intraoperatively difficult to define the boundary of intestinal ischemia. Thus, after enterectomy, anastomosis was performed, and there was an extremely high risk of anastomotic fistula. Therefore, enteral nutrition is not recommended for early postoperative patients, and the parenteral nutrition is alternatively suggested.

#### 3.3.3. Parenteral nutritional nursing

According to the expert consensus on safe parenteral nutrition infusion, the target intaking of calories for nutrition support is 25 to 30 kcal/kg/d, and the target intaking of protein is 1.5 to 2.0 g/kg/d.^[22]^ According to the “power metabolism” model, the energy requirements of critically ill patients change significantly as their metabolism changes. In the acute phase, due to increased secretion of pro-inflammatory factors (tumor necrosis factor, interleukin-1, interleukin-6) and anti-insulin hormones (cortisol, glucagon, catecholamine), they outnumber insulin, growth hormone, and testosterone. Therefore, the uncontrolled supply of heat from the outside exists the risks of matrix excess. We use international guidelines to recommend an energy requirement assessment (based on the kilogram scale algorithm). The patient’s body weight was 124 kg. The required calorie was 2480 kcal/kg/d (2.0 kcal/kg/d), and the required protein was 186 g/kg/d (1.5 g/kg/d), within 1 week of hospitalization and before anal exhaust of second surgery, in which carbohydrate ≥ 2.0 g/kg/d accounted for 50% to 70% of the total non-protein caloric calories. Fat emulsion 0.7 to 1.5 g/kg/d, and soy fat emulsion should be avoided. Because soybean oil contains a higher proportion of pure soybean oil fatty acids, it has pro-inflammatory and increased oxidative stress effects. On the 3rd day after surgery, the patient was given standardized parenteral nutrition solution infusion through central vein, and the speed of the intravenous infusion pump was controlled at 125 mL/h. The intravenous infusion of human blood ALB was performed at a dosage of 80 g/d, split into early and late transfusions. Alongside each infusion, 20 mg of furosemide was administered. The blood sugar level was measured every 8 hours to maintain blood sugar level 7.8 to 10 mmol/L. Strict aseptic technique was employed, with weekly dressing changes for the central venous catheter to prevent catheter-related bloodstream infections.

#### 3.3.4. Enteral nutritional nursing

Enteral nutrition supports the barrier and immune functions of the intestinal mucosa, emerging vital for preventing endogenous infections and acute stress-related gastric mucosal lesion bleeding.^[23]^ The patient had ileostomy manifesting on day 4 after the second surgery and enteral nutrition was subsequently started. For the patient, 500 mL of 5% glucose solution and 20 mL of 10% potassium chloride in sodium chloride were administered through the nasogastric tube at a rate of 20 mL/h for 24 hours. On the 5th day after surgery, 500 mL of short peptide-based enteral nutrition preparation (Propril) was injected at a rate of 20 mL/h for 24 hours. After increasing by 500 mL/ d, the solution was pumped at rates of 40, 60, and 80 mL/h for 24 hours. At this stage, the required calorie is added from enteral nutrition and parenteral nutrition. The tolerance of enteral nutrition was evaluated every 4 to 6 hours, and the presence of abdominal pain, abdominal distension, and the frequency and shape of stool were closely monitored. Propril, a short peptide enteral nutrient solution, is easily absorbed and effectively reduces intra-abdominal pressure and abdominal wall tension, thereby promoting the healing of the abdominal wall incision. On the 18th day after surgery, it was changed to whole-protein enteral nutrition (Nengming), containing cellulose enteral nutrition solution, which could promote the formation of stool and was conducive to the management of stoma. According to the German Sepsis Association, a nutritional support program of “less before more” should be followed. Initial calorie is added according to 10 to 20 kcal/kg/d; stable period calorie is added according to 25 kcal/kg/d; recovery period calorie is added according to 20 kcal/kg/d (obese patient). On the 22nd day after surgery, the patient’s ALB, pre-ALB, Hb, C-reactive protein, procalcitonin, the total Nutrition Risk Screening score, and body mass index were 42 g/L, 0.29 g/L, 107 g/L, below 10 mg/L, <0.15 ng/mL, below 3, and 38 kg/m^2^, respectively, and the nutritional index was significantly improved.

### 3.4. Stoma separation nursing

The incidence of post-ostomy-associated complications has been reported to be 21% to 71%.^[24]^ Fecal water dermatitis can stimulate and erode the skin around the stoma, leading to the separation of the skin and mucosa. Thin stool, which contains digestive enzymes and is weakly alkaline, can leak retrogradely into subcutaneous adipose tissue, causing stoma separation.^[25]^ This can be classified by circumferential range into partial separation, where only a part of the circumference between the skin and mucosa is separated, and complete separation, where the entire circumference is affected. Based on the anatomical depth, separation is further categorized into shallow separation, occurring in the skin and subcutaneous fat layer, and deep separation, where the depth reaches the anterior sheath of the rectus abdominis and may even penetrate the muscles and peritoneum. Depending on the wound condition, a stoma nurse can use skin care powder or appropriate dressings to create a moist healing environment. In the absence of infection, foam dressings can manage seepage, while alginate or hydrophilic fiber silver dressings can fill mucosal separations to promote granulation tissue growth.^[26,27]^ In this case, due to abdominal cocoon and obesity, ileal draw-out section was limited and the tension was large, and the separation of ileal single cavity stoma occurred on the 7th day after surgery. As intestinal fluid continued to leak into the subcutaneous tissue, the patient developed fever and elevated hemogram. A re-suture method was employed utilizing a 4-0 absorbable suture with intermittent stitching: 0.5 to 1 cm for the intestinal wall, 0.3 cm for the skin, and everted mucosal sutures spaced 0.3 cm apart. To reduce local edema, the local wet compress was applied with 3% saline twice daily. The patient was kept fasting for 3 to 5 days and treated with somatostatin to inhibit intestinal fluid secretion and reduce abdominal distension. Hypoproteinemia was corrected, and nutritional support was enhanced. Typically, the drainage strip was removed within 48 hours, the Vaseline gauze within 10 days, and the sutures within half a month. In this case, timely closure of the separated stoma controlled the subcutaneous infection, playing a crucial role in preventing incision infection and splitting.

### 3.5. Abdominal wall incision nursing

The incidence of intestinal fistula caused by severe abdominal trauma was 21.7%, and the fatality rate was 5.3% to 23%.^[28]^ Intestinal fistula may result in a series of pathophysiological changes, such as electrolyte disturbance, acid–base imbalance, malnutrition, abdominal infection, moisture-related skin injury, and organ dysfunction. The accumulation of intestinal digestive fluid in the abdominal cavity can lead to corrosion of abdominal tissues and organs, resulting in the limited or diffuse peritonitis, abdominal abscess, and potentially, erosion of blood vessels leading to abdominal bleeding.^[29]^ In the present case, on the 12th day after surgery, the proximal ileal single cavity stoma was performed in time to block intestinal fluid accumulation in the abdominal cavity, and Li’s double cannula was placed to fully drainage of abdominal cavity. The incision was defibrillated, the subcutaneous tissue was fully freed, the muscular tension of the abdominal wall was reduced, and the suture was interrupted with 4-0 absorbable thread. Below the subcutaneous layer, Li’s double cannula was inserted for intermittent irrigation and negative pressure drainage. This procedure could reduce subcutaneous fluid or blood accumulation, improving the adhesion of the muscle layer and subcutaneous tissue while eliminating potential gaps. Furthermore, the gastrointestinal decompression was continued to reduce internal abdominal pressure, and the abdominal band was limited to prevent the incision from splitting. Controlling systolic blood pressure < 120 mm Hg is conducive to reduce wound bleeding. After 72 hours, once the incision flushing liquid would become clear, the intermittent flushing with the Li’s double cannula could be stopped, and only continuous negative pressure suction was maintained. However, after 10 days, necrosis appeared around the incision, causing it to split. A doctor was consulted to remove the suture and assess the closure of the subcutaneous tissue and muscle space around the incision. The necrotic skin margin was resected, and the vacuum-assisted closure technique was employed to cultivate granulation tissue. With enteral nutrition and albumin supplementation in place, the incision healing was delayed, while reinfection or further splitting of the incision was avoided. We believe that the cases of low intestinal fistula combined with incision splitting and abdominal infection, prompt surgical intervention is necessary. This typically involves closure of the distal small intestine, creation of a proximal single cavity stoma, and abdominal irrigation and drainage. These measures constitute an effective approach to promote surgical intervention. Besides, the importance of muscle suture selection and abdominal belt application was emphasized to reduce abdominal pressure in patients with abdominal cocoon and intestinal fistula. Early-stage continuous negative pressure suction and flushing mitigate dead space, ensuring a sterile environment and minimizing incisional infection risk. Ultimately, the vacuum-assisted closure technique could promote adipose tissue granulation growth, thereby enhancing fibrous tissue healing in the incision.

In conclusion, navigating the complex management of intestinal rupture in abdominal cocoon necessitates a more efficacious approach, highlighting the importance of accumulating comprehensive nursing expertise through such cases. In addition, the patient is obese, the application of air mattress, every 4 hours to help turn over once, the sacral tail massage, can prevent the occurrence of pressure sores; The application of pressure pump can promote the venous return of lower extremity and reduce the formation of deep venous thrombosis.

## Acknowledgments

We would like to thank Prof Gu Guosheng and Ye Xianghong, Research Institute of General Surgery, Jinling Hospital, Medical School of Nanjing University, Nanjing, China, for their guidance and help to us.

## Author contributions

**Data curation:** Haibo Chu.

**Funding acquisition:** Yuxu Zhong.

**Investigation:** Haibo Chu.

**Methodology:** Yuee Hu, Yanyan Qin, Wei Dong, Haibo Chu.

**Resources:** Wei Dong.

**Writing – review & editing:** Yuee Hu, Yanyan Qin, Yuxu Zhong, Haibo Chu.
